# Computational analyses of eukaryotic promoters

**DOI:** 10.1186/1471-2105-8-S6-S3

**Published:** 2007-09-27

**Authors:** Michael Q Zhang

**Affiliations:** 1 Cold Spring Harbor Laboratory, 1 Bungtown Road, Cold Spring Harbor, NY 11724, USA

## Abstract

Computational analysis of eukaryotic promoters is one of the most difficult problems in computational genomics and is essential for understanding gene expression profiles and reverse-engineering gene regulation network circuits. Here I give a basic introduction of the problem and recent update on both experimental and computational approaches. More details may be found in the extended references. This review is based on a summer lecture given at Max Planck Institute at Berlin in 2005.

## Background

The promoter of a gene is defined as the *cis*-regulatory DNA region at a specific location (the transcription start site, or TSS) that can drive the transcription of its target gene in response to environmental signals. Computationally, it is often conveniently divided into three regions: the core-promoter (~80–100 bp surrounding the TSS), the proximal-promoter (~250–1000 bp upstream of the core-promoter) and the distal-promoter (further upstream, normally excluding enhancer or other regulatory regions whose influences are position/orientation independent). The core-promoter is minimally required for the assembly of the preinitiation complex (PIC) and can drive a reporter gene at a basal level from the TSS. The proximal-promoter often contains major *cis*-regulatory elements for driving activated reporter gene expression with some tissue-specificity. However, the distal-promoter together with distal enhancers/silencers and insulators are often necessary for accurately reproducing the endogenous gene expression patterns *in vivo*, especially for early developmental genes. Distal *cis*-regulatory elements also occur in the introns and the downstream regions, and therefore computational studies of these regions have been difficult and often limited to only the conserved sub-regions and/or regions in which functional *cis*-regulatory elements form clusters. Most of our work has been focused on 1 kb proximal-promoters (defined as -700 to +300 with respect to the TSS). We have shown that DNA motifs in this region can predict tissue-specific gene expression [1]. Computational promoter analyses usually face two related problems: the localization of the core-promoter (TSS prediction) and the identification of *cis*-regulatory elements (motif discovery). Basic computational methods have been reviewed previously [2], here I emphasize some recent developments.

## Results

### New experimental developments

One recent surprise, revealed after more detailed biochemistry studies of promoter activation, is that people have underestimated the diversity and complexity of core-promoter architecture and regulation. I refer readers to the recent comprehensive review on "the general transcription machinery and general cofactors" [3].

**Figure 1:**
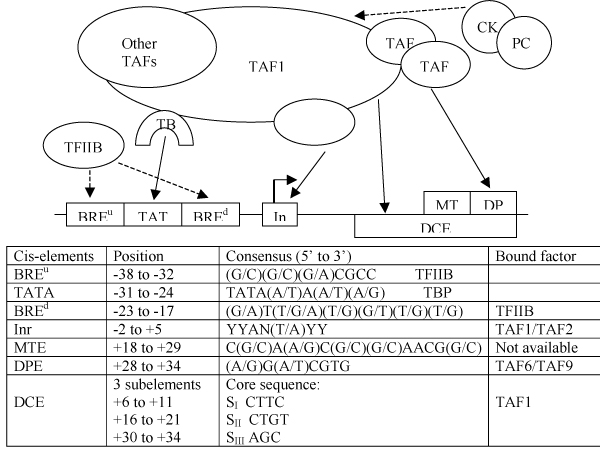
Regulation of core-promoter elements by TFIID and TFIIB (adapted from Fig. 2 of Thomas & Chiang 2006 [3]).

Although several core-promoter elements have been identified (Figure 1), with each element being short and degenerate and not every element occurring in a given core-promoter, the combinatorial regulatory code within core-promoters remains elusive. Their predictive value has also been very limited, despite some weak statistical correlations among certain subsets of the elements which were uncovered recently [4,5]. Further biochemical characterization of core-promoter binding factors under various functional conditions is necessary before a reliable computational classification of core-promoters becomes possible. An example of the type of question that must be answered is how CK2 phosphorylation of TAF1 may switch TFIID binding specificity from a DCE to DPE function [6] (Figure 1).

The most significant advance comes from the new sequencing and microarray technologies that, for the first time, can provide ample and accurate 5'UTR sequence and core-promoter/TFBS location data. In particular, large-scale 5'RACE technology at Tokyo University and 5'CAGE tag technology at Riken have provided DBTSS (Database of Transcriptional Start Sites, mainly human) [7] and Fantom (Functional Annotation of Mouse) [8,9] with an order of magnitude more promoter sequences derived from full-length 5'UTRs/cDNAs than were present in the traditional part of EPD (Eukaryotic Promoter Database) [10]. These sequences serve as the best training data for all current computational studies in promoter recognition. Many of the surprising new statistical features of the core-promoter have come from the recent analyses of such data (see [11] for a nice updated summary). One particularly interesting point made in this reference is that "Contrary to expectations, only a small fraction of RNAP II promoters appear to contain a TATA box. In contrast, a large proportion of RNAP II promoters in metazoan genomes appear to contain an INR element. Finally, about 25% of human promoters appear to lack known core promoter elements. This may point to the existence of additional core promoter sequence elements that remain to be identified and functionally characterized.". More mammalian promoter statistics are discussed in [12] which presents a comprehensive study of Fantom3 data.

In addition to sequence data, ChIP-chip technologies (e.g. see review [13]) provide genome-wide *in vivo *mapping of protein-DNA binding regions which provide the best experimental data for all current computational studies in *cis*-regulatory motif discovery. Most of the important data for promoter prediction has come from the ChIP-chip localization of PIC at active core-promoters in the whole genome at sub-100 bp resolution [14]. When more such data are produced for different tissues/cells and development stages, it will transform the field of computational promoter prediction and genome regulation networks (further discussed below).

### Advances in motif discovery

The traditional approach for finding *cis*-elements is to collect a set of (target gene) promoter sequences believed to be enriched by some common TFBS motifs. They may either be collected from the literature or from systematic experiments (such as SELEX, *etc*.). There are many *de novo *TFBS motif finding algorithms available. For a recent review on computational TFBS finding methods, see *e.g*., [15]. For a recent benchmark of some popular motif finders, see [16]. In addition to the classical alignment-based motif finding algorithms, such as CONSENSUS [17], EM [18]/MEME [19] and the Gibbs sampler [20] which have been reviewed previously [21], most modern approaches have tried to extend either to the discovery of motif combinations (called *cis*-regulatory modules or CRMs), the use of evolutionary conservation information (with either phylogenetic footprinting or shadowing approaches), or a combination of both approaches. One can also increase specificity by incorporating structural information, for example, if the protein binds as a homodimer, one could restrict the search to only the palindromic motifs.

More powerful and flexible motif finders can take the advantage of a separate sequence set called a background set, serving as a negative control. The goal is to search only for motifs that are most discriminating, *i.e*. only those enriched in the foreground set relative to the background set. Examples of such motif finders, called discriminant motif finders, include: ANN-Spec [22], DMOTIFS [23], DWE [24] and DME [25]. DME is particularly novel and powerful; it can enumerate all possible (discretized) weight matrices above user-defined minimum information content. A newer version (called DME-B [26]) of DME can optimize the classification ability of the identified motifs based on whether or not the sequence contains at least one occurrence of the motif. This technology has been used to systematically catalog of mammalian tissue-specific TFBS motifs [27,28].

The most powerful generalization of this idea would be to turn motif finding into a feature selection problem in regression analysis by asking what is the set of features ***X ***(some functions of the motifs or CRMs) that can best explain the microarray data *Y *(*e.g*. expression scores). This is very similar to the general problem in genetics: *Y *represents the phenotype (mRNA expression) and ***X ***represents the genotype (promoter DNA elements). One would like to learn a model (function *f*) so that *f(****X****) *can best predict *Y*. When "best" is measured by the average squared error based on the distribution *Pr(****X****, Y)*, the solution is the conditional expectation (also known as the regression function, see, *e.g*. [29]): *f(****X****) = E (Y| ****X ***= ***x****)*. REDUCE was the first successful motif selection algorithm based on linear regression [30]. It has now been generalized to include cross-interaction terms [31], to use nucleotide weight matrices discovered by MDscan (Motif Regressor [32]), to apply logistic regression [33] and to a nonlinear model based on regression trees called MARSMotif [34,35]. The matrix version of REDUCE (called MatrixREDUCE [36]) and of MARSMotif (called MARSMotif-M [37]) are becoming important motif discovery tools for mammalian promoter analyses. Almost all the tools developed for analyzing expression microarray data can also be easily applied to the analysis of localization data, such as ChIP-chip data. Although ChIP-chip is a global measurement for *in vivo *binding of proteins to chromatin DNA and hence is potentially capable of revealing direct target genes (most targets identified in expression arrays are not direct targets); due to the current resolution and to non-specific or non-functional cross-links, not all putative targets are functional or possess functional *cis*-elements. ChIP-chip data have also been used to further refine motifs found by expression data (*e.g*. using a boosting approach [38]).

### Better promoter prediction

A number of statistical and machine learning approaches that can discriminate between the known promoter and some non-promoter sequences have been applied to TSS prediction. In a recent large scale comparison [39], eight prediction algorithms were compared. Among the most successful algorithms were Eponine [40] (which trains Relevant Vector Machines to recognize a TATA-box motif in a G+C rich domain and uses Monte Carlo sampling), McPromoter [41] (based on Neural Networks, interpolated Markov models and physical properties of promoter regions), FirstEF [42] (based on quadratic discriminant analysis of promoters, first exons and the first donor site) and DragonGSF [43,39] (based on artificial neural networks). However, DragonGSF is not publicly available and uses additional binding site information based on the TRANSFAC database [44], exploiting specific information that is typically not available for unknown promoters.

Two new de novo promoter prediction algorithms have emerged that further improve in accuracy. One is ARTS [45], which is based on Support Vector Machines with multiple sophisticated sequence kernels. It claims to find about 35% true positives at a false positive rate of 1/1000, where the above mentioned methods find only about half as many true positives (18%). ARTS uses only downstream genic sequences as the negative set (non-promoters), and therefore it may get more false-positives from upstream non-genic regions. Furthermore, ARTS does not distinquish if a promoter is CpG-island related or not and it is not clear how ARTS may peform on non-CpG-island related promoters. Another novel TSS prediction algorithm is CoreBoost [46] which is based on simple LogitBoosting with stumps. It has a false positive rate of 1/5000 at the same sensitivity level (Zhao, personal communication). CoreBoost uses both immediate upstream and downstream fragments as negative sets and trains separate classifiers for each before combining the two. The training sample is 300 bp fragments (-250, +50), hence it is more localized than ARTS which has training sample of 2 kb fragments (-1 kb, +1 kb). The ideal application of TSS prediction algorithms is to combine them with gene prediction algorithms [21] and/or with the ChIP-chip PIC mapping data [14].

## Future direction: epigenetics and chromatin states

Although much progress has been made in promoter prediction and *cis*-regulatory motif discovery, false-positives are still the main problem when scanning through the whole genome. Fundamentally this is because the information about chromatin structure is still missing in all our models! Protein-DNA binding specificity is partly determined by the energetics and partly determined by "entropy", which depends on how much of the genome is accessible to the DNA binding protein [47] Without knowing which regions of chromatin are open or closed (and to what degree), researchers have to assume the whole genome is accessible for binding, which is obviously wrong and will lead to more false positives (and false negatives because of the extra noise). This is clearly shown by recent genome-wide ChIP-chip data as well as DNase I Hypersensitivity mapping data. There is a necessity for higher order prediction algorithms that are capable of predicting chromatin states based upon, perhaps, genome-wide epigenetic measurements, CpG-islands and repeat characteristics in addition to genomic sequences. It is fortunate that such kinds of data are rapidly being generated [48-54] and the corresponding analysis tools [55-57] are also coming along. The days of more realistic dynamic modeling of chromatin structure and its relation to expression and regulation are finally coming.
